# Maude Abbott: “A Feminine Misfit in an Exclusive Male Environment” and Her Strategies for Success

**DOI:** 10.1177/10935266241281786

**Published:** 2024-10-01

**Authors:** James R. Wright

**Affiliations:** 1 Department of Pathology & Laboratory Medicine, University of Calgary, Alberta Children’s Hospital, Calgary, AB, Canada

**Keywords:** history of pathology, Maude Abbott, McGill University, medical museums, congenital heart disease, women in medicine

## Abstract

Maude Abbott was a pioneering female Canadian physician who became a world authority on medical museums and congenital heart disease. Abbott spent almost all her career in highly sexist, discriminatory work environments. This paper reviews Abbott’s life and accomplishments, but, more importantly, analyzes her pathway to success in the masculine world of early 20th-century academic pathology. Abbott, though well-trained as a pathologist, never provided clinical service, but instead worked as museum curator at McGill University. She established the International Association of Medical Museums (predecessor to the International Academy of Pathology), edited its journal, and essentially ran the organization. Abbott, surrounded by influential males, dealt differently with each. In general, she recognized that male doctors believed women lacked the gravitas to lead major initiatives but that she could circumnavigate this supposed impediment by co-leading projects with male counterparts, preferably ones too busy to get in her way. She repeatedly used this approach, and by doing most of the work but sharing credit, succeeded in gaining reputation, accomplishment, and advancement. Abbott’s pioneering work on congenital heart disease established her as one of the founders of pediatric pathology, and, overall, her career promoted the entry of women physicians into the pathology profession.

Maude Elizabeth Seymour Abbott (1868–1940) was a pioneering female Canadian physician who became a world authority on both medical museums (collections of pathological specimens critical for medical education in the 19th and early 20th centuries) and congenital heart disease (CHD).^1-3^ While she never practiced either pathology or cardiology, she is proudly claimed by both fields. She is also recognized as one of the founders of pediatric pathology.^4^ Abbott spent almost all of her career in a highly sexist, discriminatory work environment at McGill University. During her lifetime, Abbott was treated with disdain by many McGill medical faculty and her academic efforts were sources of amusement. According to former McGill cardiologist Harold Segall (1897–1990): “In certain circles [at McGill] it was acceptable to regard Dr. Abbott as an inferior character, someone to be tolerated and humored—a “hen medic.”^5(p342)^ It should be noted that the quotation within the title of this paper was a statement^5(p341)^ from one of her few contemporary male supporters within the McGill medical school system, Charles F. Martin (1868–1953), the Medical Superintendent of Montreal General Hospital (MGH) and later Dean of McGill’s Faculty of Medicine. It was only after her death that Abbott began to be appropriately recognized at McGill.^2-6^

Many details of Abbott’s early life have been misreported. Abbott’s biographers often state she was born on March 18, 1869.^2,3^ However, her birth certificate and family census data show that she was born on March 18, 1868.^7^ Her father Jeremie Babin (1837–1913), a Church of England minister in Buckingham (Gatineau), Quebec who was widely believed to have murdered his disabled sister, abandoned his wife, Maude, and her older sister, Alice (1867–1934), likely in October 1868. Her mother, Elizabeth Bayley Abbott Babin (1840–1869), died of tuberculosis in October 1869.^8^ Abbott’s father started a new life and family in the United States. Throughout Abbott’s life, she was haunted by having been abandoned by her father as well as his scandalous past. She tried to compensate later in life by tracking down and embracing some of her many half-siblings, only to discover that her father had profoundly damaged their lives as well.^9^

Maude and Alice Babin were raised in St. Andrews East, Quebec by their maternal grandmother, Frances Mary Smith Abbott (c1808–1890). Growing up without a substantive male influence likely had implications for Abbott’s future dealings with the male authority figures she would interact with throughout her adult life (see below). The grandmother, according to legend, had the sisters’ last names changed to hers through an act of Parliament.^3,10^ However, the name change was actually done informally, as there are no parliamentary or Anglican church records of this happening (personal e-mail communication, Joan O’Malley, Administrative Coordinator, Maude Abbott Medical Museum, December 15, 2023). Regardless, the maternal side of the family tree was distinguished, as Maude Abbott was the second cousin of the third Canadian (and first Canadian-born) Prime Minister, Sir John Abbott.^2-8^ Maude Abbott, late in life, published a regional Quebec history highlighting her ancestral family’s pioneering involvement as early Anglophone settlers.^11^ Her ancestors also helped establish McGill College.^10^

From an early age, Abbott was driven to succeed.^2,3^ She was mostly home schooled but she convinced her grandmother to let her attend Misses Symmers and Smith’s School in Montreal for her last year of high school where she excelled. Nevertheless, according to McGill feminist Professor Margaret Gillett (1930–2019), Abbott’s personal diaries document that she “was not certain of her talent or her virtues” and that she “suffer[ed] from awful personal doubts as a teenager.”^12(p180)^ But, not only was she admitted to McGill University in the second year they allowed female students, she received McGill’s first ever Arts scholarship in 1885. According to Abbott late in her career: “Had it not been for this happening, I should probably not be here today, for an Arts education for a girl was at that time considered a quite unnecessary luxury. . .”^1(p128)^ Moreover, Abbott graduated as Arts valedictorian and won the Lord Stanley Gold Medal for general proficiency in 1890.^2,3^ Abbott had not initially considered applying to medical school but decided to do this during a conversation with her best friend Mary Alexandra Bell (1864–1951) [later in life a well-known artist under her married name Mrs. C.H. Eastlake who painted several portraits of Abbott].^13^ Abbott, still reflecting residual personal insecurities, had a backup plan. In addition to her B.A. degree, she simultaneously acquired “as an insurance policy, a teaching diploma from the McGill Normal School,”^12(p183)^ as this would allow her to work as a schoolteacher.

In the 1890s, only a few Canadian medical schools admitted women students, and McGill was not one of these. Nevertheless, Abbott applied to McGill Medical School; she was not admitted. However, since she was much more academically distinguished than many male applicants who were accepted, she became a focal point for gender equality with strong support from activist groups as well as negative press coverage for McGill.

Neighboring Bishop’s University, likely wanting to upstage McGill and capitalize on Abbott’s publicity, offered her a position; she enrolled in 1890. But Bishop’s had not planned carefully, as once she had completed the didactic portion with high honors, MGH (where both McGill and Bishop’s medical students did clinical work) was unwilling to let her finish her training. Because this caused more negative publicity, MGH relented and Abbott graduated in 1894 (Figure 1); upon graduation, she won Bishop’s Senior Anatomy Prize and Chancellor’s Prize based upon her final examinations.^14^ At that time, graduates interested in academic medicine completed postgraduate studies in Europe and Abbott wanted to do the same. However, her grandmother had died, and Abbott now had full responsibility for her sister, who was developing early symptoms of chronic mental illness. Abbott took Alice with her to England, Austria, Germany, and Scotland. However, Alice became increasingly ill, further complicating Abbott’s ambitious educational plans.^15^

**Figure 1.**
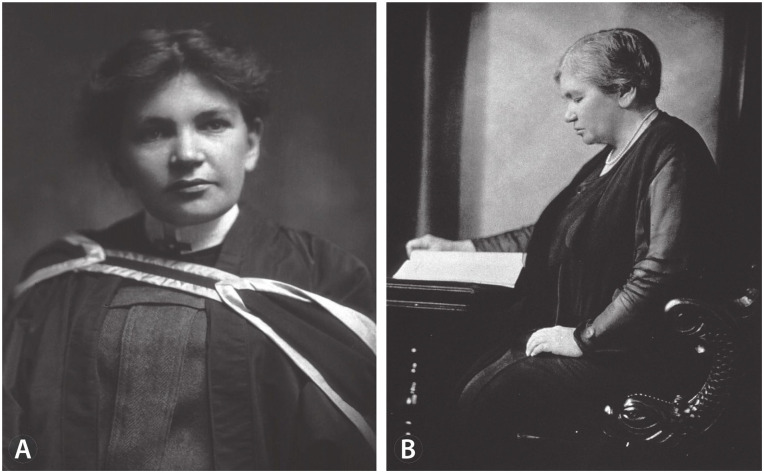
(A) Maude Abbott at her medical school graduation. NLM Unique ID: 101408316; NLM Image ID: B01504; https://collections.nlm.nih.gov/catalog/nlm:nlmuid-101408316-img. (B) Maude Abbott, c1925; Harris and Ewing photographer. NLM Unique ID: 101450958; NLM Image ID: B030311. https://collections.nlm.nih.gov/catalog/nlm:nlmuid-101450958-img. Also: McGill University Archival Resources: PR023284.

When the sisters returned to Quebec in 1898, Alice stayed in St. Andrews East while Abbott tried to earn a living in Montreal. Abbott was fully responsible for supporting Alice, providing her home care, and paying all expenses for Elmbank, the family home (https://www.communitystories.ca/v2/maude-elizabeth-seymour-abbott/gallery/elmbank/). So, Abbott, in addition to being a pioneering female doctor, was also a pioneer single professional woman with a dependent. Abbott frequently traveled back and forth to Elmbank, and her time and finances were chronically overextended.^15^ In Montreal, Abbott reportedly set up a private practice specializing in treating women and children and maintained this for many years,^2,3,6,16^ but, according to McGill Osler Librarian W.W. Francis (1878–1959): “she always had too many irons in the fire to make a financial success of it.”^16(p305)^

Returning from Europe with a love of pathology, one of these “irons” was volunteer research in the laboratory of Professor of Pathology John George Adami (1862–1926; Figure 2(a)), and she was soon assisting him with the McGill Medical Museum.^1-5,17^ Adami, the Museum director, needed someone to organize and catalog the collection of pathological specimens so that they could be used for teaching. Abbott took on a low-paying curator role. According to the standard narrative, she maintained her private medical practice to make ends meet.^2,3^ However, this may be false. Although some sources have even provided a Montreal street address, 156 Mansfield St.,^10^ for her private practice, no historical records can be found to corroborate that the practice ever existed.^7^ There is further uncertainty surrounding the dates of her curator appointments at McGill and at what point she started being paid.^7^ However, serving as an unpaid volunteer is perhaps not surprising as this is often how women in academic pathology departments elsewhere began their academic careers.^18^ Furthermore, while many authors have highlighted the degree of the disorganization of the McGill pathological museum and its uselessness for teaching prior to Abbott’s appointment, these issues have been exaggerated by both Abbott and her biographers.^7^ Nevertheless, while the early Abbott narrative contains scattered elements of hyperbole and errors, she was unquestionably the driving force converting the McGill Medical Museum into a world-wide leader.

**Figure 2.**
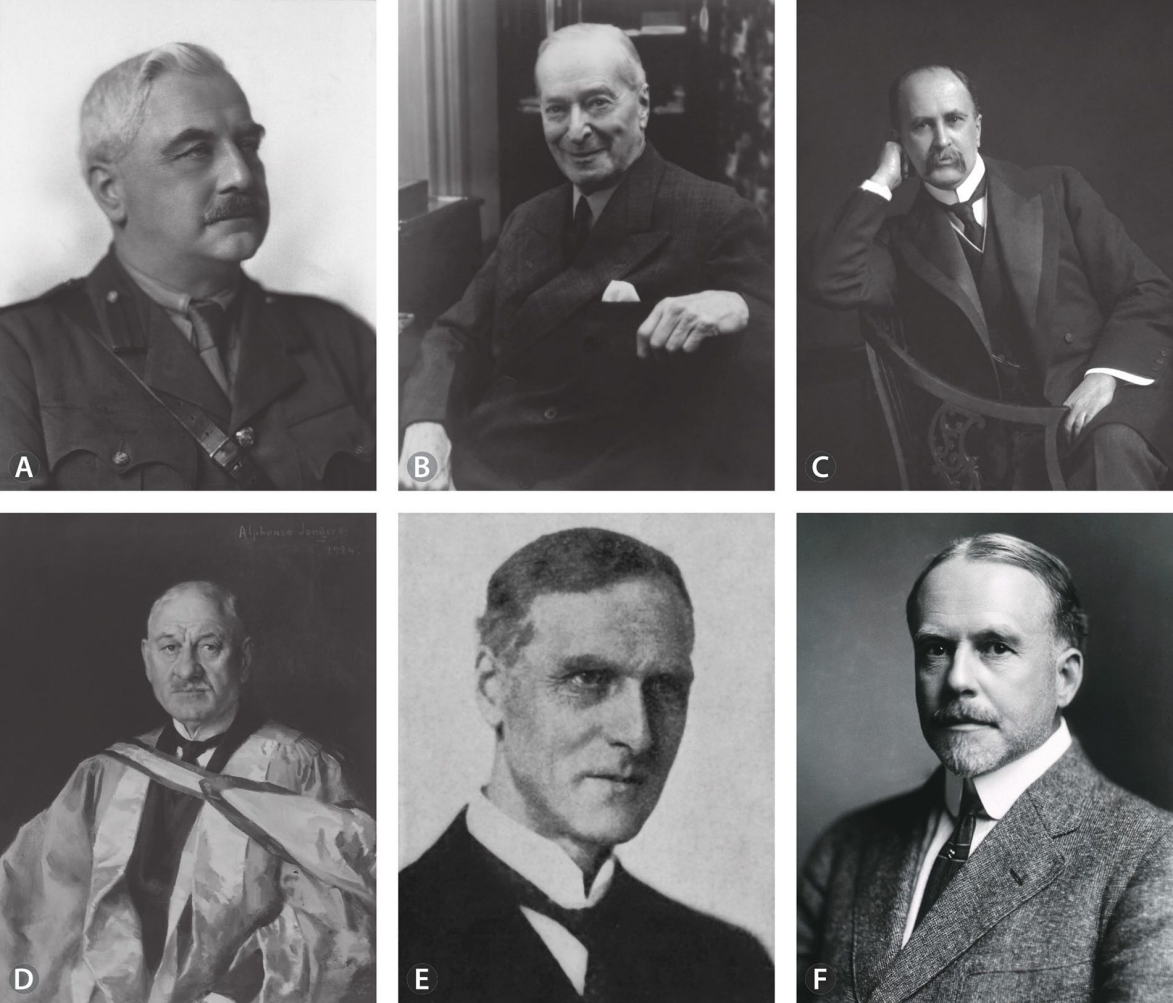
(A) J. George Adami, NLM Unique ID: 101408219; NLM Image ID: B01082 https://collections.nlm.nih.gov/catalog/nlm:nlmuid-101408219-img. (B) Charles F. Martin. Courtesy of the Osler Library of the History of Medicine, McGill University. (C) William Osler. NLM Unique ID: 101433778; NLM Image ID: B020132; https://collections.nlm.nih.gov/catalog/nlm:nlmuid-101433778-img. (D) Sir Andrew Macphail. Courtesy of the Osler Library of the History of Medicine, McGill University. (E) Sir Arthur Keith. Credit: Look and Learn. (F) Aldred Warthin. NLM Unique ID:101431578; NLM Image ID: B025337; https://collections.nlm.nih.gov/catalog/nlm:nlmuid-101431578-img.

Many of her museum specimens had been procured by William Osler, when he was the pathologist at MGH (1876–1884),^19-21^ Abbott met Osler early in her career and he became a mentor who encouraged her.^22^ In 1905, she was appointed a member of the McGill staff as a research fellow in pathology and in 1912 she was promoted to lecturer in pathology. According to a highly laudatory but chauvinist *Canadian Medical Association Journal* (*CMAJ*) obituary:

When she became Curator of the McGill Pathological Museum she restored order out of relative chaos and catalogued the specimens on a system devised by the late Professor Wyatt Johnston, a task which only the patience of a woman could face and carry out. At the same time she prepared the clinical notes which accompanied each specimen, thus facilitating greatly the studies of the students. Among the material was a fine series of hearts collected by Professor William Osler and this engaged her attention until she eventually became recognized as one of the world’s authorities on congenital heart disease. She became interested in Osler and he in her, and his inspiration became one of the driving factors in her life. One of her last activities was to prepare a complete bibliography of his works. She was a hero-worshipper of the first order and Osler was her demi-god.^23^

Throughout the first few decades of the1900s, Abbott’s stature as a world authority on medical museums and congenital heart diseases grew. In 1937, she wrote her *Atlas of Congenital Heart Disease*, which was published by the American Heart Association and is still considered an all-time classic.^24^ This was just one of her “no less than 45 articles or monographs on the subject.”^16(p306)^

According to medical museum historian, Erin H. McLeary, Abbott was the “alpha and omega of medical museums”^25(p213)^ Historian Annmarie Adams considers her to have been “the go-to expert for medical museums around the world at the height of their significance in medical education and research;” she has also been called “the mother superior of medical museums.”^26(p2)^

Abbott was a popular teacher, as her medical museum was the best way for early 20th-century McGill medical students to learn pathology. In 1901, Adami had suggested that students wanting to have museum specimens demonstrated to them make informal arrangements with Abbott. Initially, her early morning sessions were optional but soon essentially all students were participating. Because Abbott did not initially have a teaching appointment, the medical school refused to compensate her and so she volunteered her time. However, a formal letter of appreciation addressed to the Faculty from the graduating class of 1904 precipitated changes, and the sessions became a mandatory part of the curriculum.

In 1910, McGill awarded the popular teacher her first honorary degree, Doctor of Medicine and Master of Surgery *honoris causa*; this must have seemed hugely ironic to her because, as a woman, she still would not have been qualified to enroll. Abbott’s timing for obtaining a medical degree in Quebec was fortuitous, as her medical alma mater, Bishop’s Medical Faculty, closed in 1905, when it amalgamated into McGill, and the resulting merged institution did not admit women medical students.

This merger has caused historical confusion as to whether this resulted in Abbott, as well as all other Bishop’s medical graduates, automatically obtaining a medical degree from McGill, as it has been reported that the merger included a “particular proviso that its graduates were to be regarded as alumni of the McGill Medical School” and that, therefore, “Maude finally became an alumna of the Faculty which had been so ungenerous to her.”^10(p267)^ However, this is not accurate.

One condition of the merger was that McGill agreed to award *ad eundem* medical degrees to Bishop’s medical faculty members and, upon special application, some graduates.^27^ Abbott was not one of these graduates. However, a few years later, McGill awarded Abbott her honorary medical degree because of her “distinguished career as an undergraduate at Bishop’s College and for her reputation as a writer on medical subjects, especially on the subject of cardiac anomalies.”^12(p188)^ McGill was apparently unwilling to award even an *ad eundem* medical degree to a Bishop’s graduate who was a woman but did award Abbott an honorary degree.

In 1918, McGill finally opened its doors to female medical students, bowing to several decades of pressure and perhaps in part to fill vacancies left by enlisted males serving overseas. One of the woman graduates in that first class, Jessie Boyd (1894–2000),^28^ later published a biographical sketch of Abbott. Boyd wrote that “it is difficult to convey the joy and triumph of Dr. Abbott” when the 5 women graduated in 1922 and concluded “there is no doubt that her reputation had some influence on the final decision to admit women to the study of medicine.”^6^ Abbott’s pride was also demonstrated when she hired another of these 5 women, Winifred Alice Blampin (1896–1985), for her department in Philadelphia (see below).

Regardless, as noted by W.W. Francis, Abbott’s struggles had “made her an efficient champion of women’s educational rights, but politically and socially she was never a feminist.”^16(p305)^

In 1924, Abbott and 5 other Canadian women physicians formed the Federation of Medical Women of Canada (https://fmwc.ca/our-history/).^22^

During World War I, Adami enlisted and went overseas and Horst Oertel (1871–1956) served as acting chairman. After the War, Adami did not return and Oertel’s temporary role was made permanent. The new highly autocratic chairman reorganized the Pathology Department and marginalized Abbott. Oertel removed both her museum curator and medical student teaching roles. Having been demoted, she took a leave of absence in July 1923 and became professor and chair of pathology at Women’s Medical College of Philadelphia (WMCP).^29-31^ Abbott, who had previously turned down the position, now accepted it on 2 conditions—first, that she could restructure the department and second, that she could appoint her own staff (n.b., WMCP also reportedly doubled her McGill salary). Although Abbott transformed her new department, she could not stay in Philadelphia as her sister could not leave Elmbank. Abbott returned to McGill after 2 years, but before leaving, she selected her own replacement, Helen Ingleby, MB (1887 -1973), 1 of 4 women who had been allowed to study at the prestigious (and previously all male) St George’s Medical School in London during the War^32^; Abbott likely viewed Ingleby as a kindred spirit, as St George’s next hired her as its medical museum curator. Uniquely, Abbott succeeded in building a solid all female department (i.e., pathologists and technical staff) at WMCP; with the hiring of Ingleby, this persisted even after her departure. Eventually one of the pathologists Abbott had initially hired relocated to Calgary, Alberta.^1,33^

Upon Abbott’s return to McGill in 1925, she was promoted to assistant professor. However, she was no longer appointed to the pathology department. After returning from WMCP, Abbott was made curator of a small “Central” Medical Museum, but it was eventually decided that having 2 different medical museums containing pathological specimens was problematic, so in 1932, Abbott’s Museum was renamed the Historical Medical Museum. The Historical Medical Museum was mostly comprised of teaching specimens remaining from Osler’s time, the Canadian Medical War Museum specimens (see below), wax models depicting skin diseases, a collection of old medicine containers, and importantly Abbott’s CHD cases, thus allowing her to continue this research; however, her primary research arena became medical history and she reported to a medical historian, J Andrew Macphail (1864–1938).

Despite her accomplishments and increasing international acclaim, it had taken ~15 years at McGill for her to be appointed as a lecturer, their lowest faculty rank, and she was only an assistant professor when she retired. As a further indignity, Abbott, although well-trained in pathology, never performed any clinical service work or autopsies at any McGill teaching hospital. While extant historical records do not directly address whether she was actively blocked by male pathologists or whether it was simply understood that this door was tightly closed, even without these normal opportunities, with the exception of Osler, she is unequivocally the most important pathologist in the prestigious history of McGill University. A recent book entitled *Pioneers in Pathology* features only 3 Canadians: Osler, Abbott, and William Boyd. Of these, only Abbott was both born in and practiced primarily in Canada. This book also included chapters for only 7 women (as opposed to ~190 men); of these, Abbott was chronologically the earliest.^34^

Carlotta Hacker aptly summarizes Abbott’s lack of respect at home as follows:

By 1908. . ., Dr. Abbott had gathered a large following of admirers. But her reputation was greater in other countries than it was at home. At McGill, most people thought of her, rather tolerantly, as a form of female absent-minded professor, a ‘character’ who worked in a mess—papers everywhere – who dropped handkerchiefs and notebooks as she walked along, who never could find her train ticket and who, in the stress of the moment, might appear with her skirt on back-to-front or her hair all blown about. She was the soul of kindness, nobody could deny that, but she was considered an amusing character rather than a celebrity. . . on the whole McGill didn’t realize what a remarkable figure it had in its midst.^35(p163)^

## General Thoughts About Abbott’s Biographies and Biographers

Much has been written about Maude Abbott and her career, but most writings have heavily relied upon 3 sources: Abbott’s brief autobiographical paper^1^ which was written in 1928 and published posthumously in 1959 and 2 longer biographies: Hugh E. MacDermot’s *Maude Abbott*—*A Memoir*, which was published in 1941,^2^ and Douglas Waugh’s *Maudie of McGill: Dr. Maude Abbott and the Foundations of Heart Surgery*, published in 1992.^3^ While both biographies provide summaries of her life events, as highlighted above, they do contain factual errors.^7^ As correctly noted by Adams: “This heavy reliance on 2 works has meant that the same narrative has been repeated in each subsequent biographical account of Abbott’s career.”^26(p3)^ While numerous brief biographical sketches also suffer from this limitation,^8,36-38^ obituaries written by close colleagues exist providing additional perspectives.^16,39,40^ Essentially, none of these sources provide much insight as to her inner workings. However, it should be noted that additional important sources, Abbott’s diaries, personal papers, and correspondence, do and are held at both the Archives and the Osler Library at McGill University. These are potential sources of further insights.

Adams suggests that there are problems inherent in the perspectives that male biographers bring to assessing the struggles of women in the past:

The existing biographies of Abbott are textbook examples for feminist critiques of the genre. Nearly all of them try to fit Abbott’s non-conventional life and career into a traditional, biographical arc inspired by men’s life stories. Biographers focus on her struggle, on what she couldn’t do, rather than how she managed her accomplishments.^26(p2)^

According to Adams: “The existing biographies of Abbott . . . imply that Abbott was completely passive in the way her life unfolded. Writers see her life story as wholly shaped by a series of constraints and by decisions made by others. . .”^26(p4)^ Adams continues:

Like many other women in medicine and science, Abbott’s story has been told through narratives of constraint, as if all of her life choices were determined by a series of doors closed by others . . . Every account, for example, includes the story of Abbott’s rejection from McGill University on account of her gender, and her subsequent career as a museum curator as a result of her exclusion from hospital work. . . Almost all of the biographical accounts include the university and the hospital as a series of ‘closed doors’ to Abbott. In addition. . . her apparent conflict with pathologist Horst Oertel essentially ended her brilliant career as a museum curator. In such accounts, then, these pivotal movements in Abbott’s life are determined by the action of men. . . Although many of Abbotts’ major choices, then, appear to have followed on the heels of invitations offered by men, her life story offers plenty of instances of independent action. Abbott willingly took up challenges with alacrity and made projects her own.^26(p4)^

## Abbott’s Strategies for Success in a Man’s World

So, how did Abbott open doors and make projects her own? How did Abbott succeed as a female pathologist working in a man’s world, as powerful men were clearly gate-keepers? The remainder of this paper focuses on these topics in order to challenge the narrative of constraint. While it is impossible to know exactly what was going on in Abbott’s mind, an analysis of reported behaviors and behavioral patterns might suggest the ways Abbott navigated the sexist workplace and managed her interactions with male superiors.

### Good Boss, Good Employee

The analysis begins with an attempt to understand Abbott’s experience with her first boss, Adami, which allowed her to thrive. Our best insights into Abbott’s working relationship with Adami can be obtained by reading Abbott’s contribution to *J. George Adami: A Memoir*, which was edited by his wife and published in 1930.^17^ Abbott, who consistently wrote positively about powerful men who facilitated her career,^1^ credits Adami for recognizing:

Coming fresh from the old land, and familiar with the great pathological collections of the British museums, himself imbued with the spirit of the collector, and endowed with that vision of the principles of Pathology as the underlying basis for the teaching of clinical medicine that was to us one of his greatest assets, Professor Adami saw clearly the importance of conserving and developing to the full and precious legacy that lay concealed in this original collection, to which his new department had fallen heir. . . For the fulfillment of such an undertaking two steps were necessary: – (1) From the great hospitals of the School a wealth of specimens must be added, with natural colours preserved by. . . newly discovered methods. . . and carefully selected to the end that a teaching collection might result illustrating all sides of the great subject of Human Pathology; and (2) a Medical Officer with enthusiasm and energy must be appointed for the care and upkeep of the Museum, to whom might be entrusted the conservation, development and cataloguing of the collection. [specifically, Adami and Abbott agreed] “the Museum needs a lover”`^17(p151-152)^

But, little happened until Abbott returned from 3 years of post-graduate study in Europe, and began volunteer work in Adami’s laboratory. Abbott quickly impressed him with both her knowledge of pathology and her work ethic. Later that year, the Faculty appointed her “Curator of the Medical Museum, on the recommendation and under the Directorship of Professor Adami”^17(p153)^ [n.b., elsewhere Abbott states she was appointed assistant curator in 1898 and curator in 1899,^1(p141)^ but this is the timeline Abbott provided in the quoted source]. From this grew a system of specimen classification, a descriptive catalogue, and a system for small group teaching of medical students. Abbott also credits Adami for her “organization of the International Association of Medical Museums, an undertaking carried out in collaboration with the Curator of the Army Medical Museum under Professor Adami’s personal supervision and interest.”^17(p153)^ Abbott credits “Professor Adami’s generous and kindly administration” for the transition of “a chaotic mass of material in an indifferent state of preservation. . . [into] one of the first Teaching Museums on the continent.”^17(p154)^ She credits this transformation primarily to:

Professor Adami’s mental attitude, his scientific breadth of vision, and his generosity of spirit to those in his Department. He was at all times a sympathetic and inspiring Chief, unfailing in his interest and encouragement in the work at hand, and to me, in my work in and for the Museum, he always gave that best of guerdons, freedom for development of my own ideas and ideals in what had become my chosen field of labour, and that autonomy in the management of details that is a necessary condition for sincere and honest work.^17(p154)^

Clearly, supportive oversight, a high degree of independence, and a strong work ethic were a recipe for success while working with Adami who provided a positive work environment.

#### Abbott and Martin

Other than Adami at the beginning of her academic career, Charles F. Martin was the most important male figure with whom she needed to interact, as he was hugely influential and eventually Dean of Medicine at McGill; Abbott periodically needed his help. According to her biographer Waugh, “although Maude’s hero was Sir William Osler, it was to Dr. Martin that she most frequently turned for support and advice on personal matters. They were almost contemporaries but in his frequent letters to her Martin’s tone was often that of a doting parent offering praise and encouragement or chiding her for being too emotional.” Indeed, according to Abbott’s friend, cardiologist Harold Segall, “Whenever she burst into tears he [Martin] would console her and she got what she wanted.”^3(p83)^ According to Waugh, Martin’s remonstrances about her being too emotional were tempered by his assurance that emotional fragility was a source of a woman’s charm provided, he admonished, it was properly directed.^3(p113-114)^ Abbott does not appear to have displayed “emotional fragility” with other senior males.

Martin was sympathetic to Abbott as, since her medical student days, he had witnessed firsthand the discrimination and persecution she had suffered and overcome. It should also be noted that Martin jumpstarted Abbott’s career in 2 ways. First, he had supervised her for a successful research project on heart murmurs shortly after she had returned from her European studies and then, next, knowing of her interest in pathology, he introduced her to Adami. In her Autobiographical Sketch, she writes that Martin “has always been my best friend in the University.”^1(p152)^

### Asymmetrical Co-Leadership

By early in her medical career, Abbott had gained good insights into the inner workings of the male medical hierarchy and began adapting, actively facilitating her own advancement. For instance, she appears to have recognized that, as a woman, male doctors believed that she lacked the gravitas to lead major medical initiatives but recognized that she could circumnavigate this supposed impediment by co-leading projects with a male counterpart, preferably one who was too busy to get in her way. As documented below, she repeatedly used this approach, and by doing most or all the work, it was almost like being fully in charge; and in some instances, she eventually ascended from *de facto* leadership to full leadership. Likely, her unpaid work experience with Adami became the initial prototype for this approach. Abbott had started as a volunteer at the McGill Medical Museum but, because of her success in this role soon became its Curator with Adami as its Director. Not only was this an “open door,” through hard work and diligence, it led to an entire career pathway.^26^ However, this approach required nuanced modification when dealing with other male leaders.

#### International Association of Medical Museums and its *Bulletin*

When dealing with men who were not as trusting or as willing to relinquish control as Adami, Abbott would volunteer to take on important tasks with 1 or more busy, prominent male colleague(s); even though formally working in a co-leadership model, she would shoulder most of the work and then share the credit. For instance, she was a co-founder of the International Association of Medical Museums (IAMM) and co-editor of its journal.^41^ Both the Association and its journal thrived and, as a result, she became internationally recognized as an expert in all aspects of medical museum work. According to Robin A. Cooke, Abbott was the founding Secretary and Treasurer of the IAMM in 1906–1907 and served in these roles until her death; she was 1 of 5 founding office bearers and 1 of 44 founding members of the IAMM. “William Osler suggested that the Association should publish a regular News Bulletin to record its proceedings, and to provide information regarding the exchange of specimens between museums.”^41(p70)^ Cooke notes that IAMM *Bulletins* No. I (1907), No. II (1909), and No. III (1910) had no named editor but concluded they were clearly written by Abbott. *Bulletin* No. IV was published in 1913, with University of Michigan chair of pathology Aldred Scott Warthin (1866–1931)^42^ and Abbott listed as co-editors (n.b., tellingly, they were not listed in alphabetical order). By the time of *Bulletin* No. VII (1918), the *Special War Bulletin of the American and Canadian Section*, 4 editors were listed: Warthin and his junior associate Carl Vernon Weller (1887–1956) from Ann Arbor, MI, and Abbott and her junior associate Louis Gross (d1936) from Montreal.

Bulletin No. VIII, which appeared as a massive 633-page hardcover book subtitled the *Sir William Osler Memorial Number*, published in 1926, listed 6 exceedingly well-known North American pathologists, including Warthin, as its Editorial Board with Abbott listed underneath as “managing Editor.”^43^ Waugh documents that the book was her idea, that she convinced the IAMM board to agree to the massive undertaking, that she establish the editorial board comprised of “the Giants of North American pathology," that she raised approximately $3,000 of funding for the book by identifying “subscribers,” and that she solicited the authors for the 119 articles within. “Not only did she solicit contributions, she . . . [refused] to take ‘No’ for an answer from those who said they were too busy.”^3(p88-89)^ The entire project took her 6 years to complete. This is clearly another prime example of taking on an important task, one that began solely as her idea, with busy, prominent male colleagues, but shouldering almost all of the work and sharing some of the credit.

After 27 years of being the *Bulletin*’s driving force, Abbott was finally listed as its “Editor” when *Bulletin* No. 13 was published in 1934. With No. 17 (1937), Abbott, likely for the purpose of succession planning, gained an Associate Editor, Robert Moore of St. Louis. With No. 18 (1938), Moore was Editor and there was a special tribute to Abbott who had retired.^41^ While Abbott’s biographers always highlight that she was a co-founder of the IAMM and the editor of its *Bulletin*, none have documented how long it took her to obtain the actual title of editor. Other than secretary-treasurer, she never held any other office in the IAMM. During her membership, there were 17 presidents, including her *Bulletin* co-editor Warthin—twice. Likely secretary-treasurer, was deemed to be an appropriate gender-based role during this timeframe; she also functioned as an unofficial historian and was posthumously honored for having kept a scrapbook detailing the early history of the IAMM.^44^

Several decades after the IAMM transitioned into the International Academy of Pathology (IAP) and 4 decades after her death, the organization realized what an asset she had been all along, fully embraced the importance of her roles, and established an annual Maude Abbott Lecture (https://www.uscap.org/maude-abbott-lecture/) in 1980.

##### Canadian Medical Association Journal

During World War I, an opportunity that normally would have only been open to a male physician materialized as most had enlisted and gone overseas. Abbott, now an experienced journal co-editor, served as the “Acting Editor” of the *CMAJ* throughout the War. According to Abbott in her Autobiographical Sketch:

. . .nearly everyone we cared for [i.e., her male physician colleagues at McGill] went across. My part was of course to carry on at home, and I was given the Acting Editorship of the Canadian Medical Association Journal, and did my best to keep it from going under during that troubled and short-handed time.”^1(p149)^

Abbott’s wording is not precise as the founding editor J. Andrew Macphail enlisted, went overseas, and earned a knighthood, but never actually resigned as editor until after the War. Abbott and George Gordon Campbell (1863–1932) *functioned* as co-acting editors throughout the War but were never officially given titles. Little is known about Campbell (1863–1932); according to a brief obituary by Albert George Nicholls (1870–1946), *CMAJ* Editor from 1930 to 1942, Campbell was “connected with the teaching staff of McGill University, and was in charge of paediatrics and dermatology at the Montreal General Hospital.”^45(p181)^ He also noted:

During, the Great War, while the editor of our *Journal*, Sir Andrew Macphail, was overseas, Doctor Campbell, together with Dr. Maude Abbott, acted in his stead. At that time grave doubts were expressed as to whether the *Journal* would be able to carry on under the adverse conditions then pertaining in the profession. Doctor Campbell and Doctor Abbott, with quiet, determined optimism carried the thing through, and we owe them a great debt of gratitude for the part they played at that crisis in our history.^45(p181)^

Abbott out-lived Campbell, but in her *CMAJ* obituary,^23^ there is no mention of her role in keeping the *Journal* afloat during the War. Regardless, they had shepherded a 3-year-old journal through the War as unofficial acting co-editors and then turned it over not only unscathed, but actually stronger, to a new editor, A.D. Blackader, as soon as Macphail retired from this role. Unfortunately, the *CMAJ* website, which lists the *Journal*’s former editors-in-chief, does not mention Abbott and Campbell with even an asterisk (https://www.cmaj.ca/page/staff). Parenthetically, while the website lists the brief service of two female “interim” editors-in-chief (both appointed after 2006), it took 110 years for the *CMAJ* to appoint its first female editor-in-chief.

The fourth *CMAJ* editor-in-chief (1942–1955) was Hugh Ernest MacDermot (1888–1983), who also authored the first of Abbott’s 2 primary biographies. MacDermot barely mentions Abbott’s unofficial acting role in the biography, simply stating that “the Journal had only just begun to get into its stride when the war absorbed its editor, Dr. Andrew Macphail, and his assistant, Dr. W.W. Francis.” He then noted that “Maude carried on much of the editorial work under very difficult conditions” and that “she received no remuneration for this work.”^2(p117)^ MacDermot does not even mention Campbell; likely he recognized that Abbott had carried most of the load relative to her male counterpart, who had had substantial clinical duties at MGH further exacerbated by understaffing because of the War. This appears to be another prime example of Abbott taking on a co-equal role with a busy male physician and then doing most of the work.

#### Abbott and Macphail

Macphail^46,47^ and Abbott interacted throughout much of their lives. They were undergraduate students together. Macphail completed his Bachelor of Arts degree in 1888. Macphail received his medical degree from McGill in 1891. Throughout his studies for both degrees, he was preoccupied with writing for the *Montreal Gazette* and was an accredited correspondent for the *Chicago Times*. As a result, he was both busy and an average student. Abbott, on the other hand, was at the top of her class at both McGill and Bishop’s. Macphail was appointed professor of the diseases of children at Bishop’s Medical College in 1893, the year before Abbott graduated. He was appointed a consulting pathologist at Montreal’s Western and Verdun Hospitals in 1895; in contrast, Abbott returned to Montreal from her postgraduate European studies in 1897, which had included extensive training in pathology, but never practiced pathology in Montreal. In 1907, Macphail was appointed chair of the history of medicine at McGill, a position he held until his death in 1938. Later in her career, Abbott reported to him in that role. Also in 1907, Macphail began working with the Canadian Medical Association to produce a journal; in 1910 he was appointed its first editor and in 1911, the first issues of the *CMAJ* were published.^46,47^ As noted above, Macphail enlisted in 1914 and served with distinction in Europe until the end of the War without actually resigning. According to an article published when Macphail’s successor retired in 1929: “While the Editor, Sir Andrew Macphail, was away on active service the Journal was capably administered by Drs. Maude Abbott and Gordon Campbell. After the war Sir Andrew resigned and an Editorial Board was constituted, with Dr. Alexander Dougall Blackader (1847–1932) as Chairman. This was in 1919.”^48(p367)^ A review of Abbott’s professional correspondence in Maude Abbott fonds (https://archivalcollections.library.mcgill.ca/index.php/maude-elizabeth-abbott-fonds) does not provide any evidence that Macphail advised Abbott while she was capably co-administering the *CMAJ* (i.e., while he was absent as editor-in-chief). Likely, Abbott had observed that Macphail had little interest in overseeing her activities, and so, Abbott decided she could simply ignore him and go directly to the more sympathetic and malleable Martin, when in later life she reported to Macphail at McGill.

### Dealing With Closed Doors

In general, Abbott appears to have shrewdly avoided doors that were likely impossible to open and focused on ones that could be pushed open just wide enough for her to squeeze through. For instance, the author and other Abbott scholars have found no compelling evidence that she fought to do clinical work in pathology or to do autopsies at the McGill teaching hospitals; likely because she was fully aware of the strong resistance she would face from her medical school experiences and the fact that, with the exception of Martha Wollstein, who because she was independently wealthy and could be hired inexpensively by an exceedingly frugal medical director at Babies Hospital in New York City,^49,50^ there were no North American women practicing as hospital pathologists at the beginning of the 20th-century.^18,51^ Instead, she more strategically created a niche for herself in the medical museum arena.

#### Abbott and Keith

Abbott played a series of secondary roles, but while doing essentially all the work, in the development of the Canadian Medical War Museum (CMWM).^52^ Briefly, Abbott wanted to support the war effort medically, but there is no evidence that she tried to enlist. Invoking once again Adams’ door analogy,^26^ Abbott likely recognized that as a woman physician, this door was tightly shut. Instead, she agreed to serve from Montreal as a museum expert helping build the CMWM, a collection of instructive pathological specimens derived from soldiers dying of battle wounds, chemical warfare, infectious “camp diseases,” and influenza. In this instance, since she was not enlisted, she could not serve as its medical director. Even though she was recognized as a world expert, the Royal Canadian Army Medical Corps, because of high turn-over, sequentially appointed a series of 5 enlisted male medical officers with no expertise in pathology or interest in medical museum work as CMWM director. She and her technical staff did the vast majority of the work, as they were responsible for preparing specimens for display, occasionally displaying them at medical conferences, and then protecting them until a museum could be created after the War.^52^

The upstream collection, preservation, and distribution of pathological specimens for all Commonwealth countries, including Canada, was being overseen by Arthur Keith (1866–1955), the famed British anthropologist and conservator of the Hunterian Museum at the Royal College of Surgeons (RCS) in London.^53^ Canadian pathologist Morton E Hall (1887–1975) was assigned to RCS to assist Keith with museum specimen preparation.^54^ Therefore, one of the roles Abbott could have fulfilled was already taken by a highly qualified enlisted pathologist. So, Abbott proposed that a knowledgeable pathologist was needed upstream to serve in Canadian field hospitals to optimally collect, preserve, and ship specimens from France to the RCS, and she desperately wanted that position.

During the War, specimens were being shipped sporadically from Keith to Abbott at McGill for inclusion in the CMWM collection. Abbott wanted more active involvement, and so she wrote Keith asking his permission to go overseas to help with the collection of pathological specimens for the War Museum. Because of its high illustrative value, this letter is quoted at considerable length:

The MEDICAL MUSEUM

McGILL UNIVERSITY

Montreal August 6th.18

Dr. Arthur Keith,

Royal College of Surgeons, London, Eng.

Dear Dr. Keith:

Thank you so much for your most kind letter. I am very glad to hear that a man [Lloyd Phillip MacHaffie (1889-?)] has been appointed for France. . . We will look eagerly for results. . . I am of course much disappointed however, that you feel you do not need me [in either London or France]. Dr. Adami tells me this is partly because you are shorthanded at the College and could not give me the assistance there that you think I would need. But in my present offer I did not have any thought of doing work at the College on the Specimens that are there. What I offered myself for was to assist in getting the work going in France, to "back MacHaffey" [sic] in the sense of drawing up a plan of action for him to follow, which in its turn would be officially backed by you and Dr. Adami. This would not need assistance other than stenographic and that supplied by Dr. McHaffie himself. Together he and I would do for the Canadian specimens what you say Dr. Elliott did for the British. I do not honestly see how a really junior man as he is can organise things to the maximum degree of efficiency in France . . . [Together] we surely could get ideal results, or results at least as good as if a senior man and pure pathologist . . . were appointed. This would only be using me in an advisory or consultant capacity which my past experience justifies. . . the very essence of my work has been the organization of Museum collections, and the magnitude of the results to be obtained for the War Museum are so self evident, and the other hand the bringing me across as a civilian for a few months is such a little thing for everyone except myself, that it seems to me some misunderstanding must lie behind your refusal of my services in this connection. Thus you say I am more needed here for the War Museum. But for the short time I proposed to go, the work here could not miss me at all, and my return passage to Canada could be booked a month before I sail, to avoid undue delay in returning. . .So urgent did the need of my services there seem to me and so complete was my faith in the support which the giving of this opportunity to me would be sure to find with you and Sir William [Osler] and Dr. Adami that I never dreamed of a refusal and was making all sorts of arrangements. . .[Abbott then explains how her trip could be, for the record, arranged by her through the IAMM and that her business trip could then] be utilized for the Canadian specimens too. . .I will write you later about this. Meantime dear Dr. Keith please forgive this long discussion of your decision, and in the second place I beg you to please keep my offer of my services there before your mind, so that, if the required degree of efficiency is not attained in the present arrangements, you may feel able to invite me to come over and help a little later on.

Yours very truly,

Maude Abbott^55^

Abbott’s letter utilizes many persuasive arguments and then asks Keith to reconsider “if the required degree of efficiency is not attained.” In her follow-up letter dated September 7,1918, she immediately describes some process inefficiencies that may have resulted in the loss of 52 “very beautiful specimens” [she provides a list with identifying #s, soldier’s names, and very brief descriptions]. She concludes with: “We hope for a fresh consignment of War specimens soon—with records, if possible, through Dr. McHaffie’s efforts!”^55^

Both letters, while respectful to admired males with pathology museum content expertise, barely hide her contempt for McHaffie, whose name she sometime misspells. Sadly, her unequivocally superior qualifications were being trumped by his gender and there was simply no way she would be going to either England or France as part of the war effort. Unfortunately, Abbott’s papers at McGill do not include correspondence with Keith expanding on this particular request. However, Abbott did discuss her interest in going to England with Adami, who was not supportive and suggested she drop the idea.^3(p83-84)^ In this instance, Abbott had clearly miscalculated, as she had repeatedly pushed on a door that would not budge.

### Supportive Peer and Mentor Relationships

Adams has shown that 1 key to Abbott’s success was developing and maintaining a vast network of friends. Through a detailed analysis she has described as “friendship archeology,” she examined Abbott’s friendship with 2 highly influential male cardiologists, Emanuel Libman (1872–1946; eponymously remembered for Libman-Sacks endocarditis) and Paul Dudley White (1886–1973; eponymously remembered for Wolff-Parkinson-White syndrome); these relationships were clearly symbiotic.^56^ These men were colleagues and she did not report to either of them. Furthermore, her friendship with both evolved later in life when her career was already established. Nevertheless, both relationships were important. Libman, who was extremely wealthy, became a friend who helped her financially with her research and book writing efforts and, since she was occasionally strapped for cash, sometimes even gave her money to help with personal expenses. Libman was so influential that in celebration of his 60th birthday, Abbott and over 140 of his other friends generated a magnificent ~1300-page-long, 3 volume book set in his honor—each friend providing an original scientific contribution. Abbott’s paper was entitled “On the relative incidence and clinical significance of a congenitally bi-cuspid aortic valve.”^57^ White wrote the forward for Abbott’s *Atlas*^24^ and also published his reminiscences of Abbott after her death.^39^

#### Abbott and Osler

Abbott’s face-to-face interactions with William Osler were limited but she liberally credits him with mentorship and advice that guided her career.^1,16^ Osler left McGill ~14 years before Abbott’s curatorship appointment. Her earliest exposure to him was when she attended his hospital rounds at Johns Hopkins when passing through Baltimore during a trip to study the organization of specimens at the Army Medical Museum in Washington, D.C. She was part of a large group and might not have been noticed had she not gotten the tip of her finger crushed in a door. While Osler tended to the injury, she took the opportunity to tell him about her new role at the McGill Medical Museum, which was, of course, of tremendous interest to him as he had collected many of its specimens while working as the MGH pathologist. Osler then invited her to join a group he was assembling for dinner that night, which she accepted, and this facilitated additional discussion, which she always credited thereafter with clinching her decision to devote her life to medical museology. Abbott also credits Osler with advice on forming the IAMM, another career-defining decision. While the finger injury may have been, as described by Abbott, “an unpleasant, but certainly fortunate, accident,”^2(p72)^ it created an opportunity which she seized. Perhaps strategically, she does not bother to mention meeting Osler via a finger injury in her Autobiographical Sketch.^1^ Regardless, she must have recognized that embracing Osler as her personal mentor created the opportunity for her to “name drop.” Furthermore, possessing a close linkage with Osler potentially allowed her to borrow his gravitas to help her open doors. For instance, her close relationship with Osler was one of her primary bonds with Libman.^56^

Adams notes that:

Abbott herself used highly charged language to describe the famous physician’s influence on her career. In a much-cited quote from a fateful dinner of Abbott and Osler in Baltimore in December 1898, Abbott engages terminology from reproduction and fertility to illustrate his ‘seminal’ role. . .”^26(p6)^

As Abbott was writing her Autobiographical Sketch in 1928, she concludes with brief statements of appreciation to several men who helped her; she ends this with:

And last but not least, for Sir William Osler, whose keen interest in my work and broad human sympathy pierced the veil of my youthful shyness with a personal stimulus that aroused my intellect to its most passionate endeavour.^1(p152)^

The sensual nature of Abbott’s descriptions has been attributed by both male and female biographers to hero-worshiping tendencies. For instance, even staunch feminist McGill historian Margaret Gillett notes “she was something of a hero-worshipper. Pre-eminent among her heroes was Sir William Osler. . . It may not be stretching too far to place a Freudian interpretation on her account of her first meeting with Dr, Osler.”^12(p190-191)^

To provide additional context, it is worthwhile digressing momentarily to examine Osler’s advice to other young women physicians or trainees. First, it should be recognized that Osler took a pragmatic approach to women in medicine. While his writings show occasional examples of sexism, especially when viewed through the 21st-century lens of “presentism” (judging historical figures against modern-day values not generally embraced at the time they lived)^58^ his reservations tended to be more economic than ideological. For instance, in his 1885 presidential address to the Canadian Medical Association, he said:

It is useless manufacturing articles for which there is no market, and in Canada the people have not yet reached the condition in which the lady doctor finds a suitable environment. Look at the facts as they are: even the large cities can support only one or two. . . and in the smaller towns and villages she would starve. . . Do not understand from these remarks that I am in anyway hostile to the admission of women to our ranks; on the contrary, my sympathies are entirely with them in the attempt to work out the problem as to how far they can succeed. . .^59(p847)^

However, unfortunately, Osler’s supply and demand argument was quickly embraced by McGill Principal Sir William Dawson (1820–1899) as another reason for excluding women students from its medical school.^14^

A few years later, shortly after his appointment as professor at Johns Hopkins, in the context that the medical school could not open its doors without donations from wealthy local women who were insistent that women be admitted on an equal basis as men, he became “warmly in favor” of co-education.^59(p848)^

Like Abbott, many women physicians and medical students found Osler supportive; he would listen and offer personalized career advice. In general, Osler believed woman physicians would most likely find success treating women and children, as missionaries, and “in sciences such as bacteriology, histology, and pathology.”^59(p848)^ One hundred plus years ago, this was sage advice and consistent with his advice to Abbott. However, no other female physician seems to have embraced Osler in quite the same way as Abbott did—essentially not only as a helpful colleague, but also as a personal muse and mentor.

After their accidental introduction, Abbott was always motivated to impress Osler, and Osler greatly admired the quality of Abbott’s work. When he had asked her to generate a chapter on congenital heart disease for his multivolume *Modern Medicine its Theory and Practise*,^60^ he was delighted with the final product, writing:

I knew you would write a good article but I did not expect one of such extraordinary merit. It is by far and away the very best thing ever written on the subject in English – possibly in any language. I can not begin to tell you how much I appreciate the care and trouble you have taken. . . For years it will be the standard work on the subject. . .^1(p146)^

It was undoubtedly positive for Abbott’s personal reputation, though sad from a present-day perspective, that her contribution was the only 1 (out of 104) authored by a woman.^22(p348)^ By the time of publication of Osler’s and McCrae’s third edition in 1927, Abbott’s chapter had grown to 200 pages in length.

Clearly, Abbott was so proud of Osler’s letter that she “carried that glowing tribute with her in her handbag”^12(p186)^ for so long that it was beginning to deteriorate and so the “curator of the Osler Library had it bound for her on the condition that she leave it to the library”^12(p419,fn12)^

Related to Abbott’s organization of the Museum and its incorporation into teaching at McGill, Osler wrote from Oxford in 1905: “Dr. Abbott’s collection grows apace – it will be one of the best pieces of work ever done at the school. . . I do not believe there is a museum in Great Britain with a better collection – there is nothing like it on this side.”^5(p335)^

Likely, Osler’s most important influence on Abbott’s career was her linkage with him, which she repeatedly proclaimed, and not necessarily occasional bits of advice on museology. Adami’s advice, by virtue of his day-to-day availability and his strong personal vested interest to reinvigorate the Museum under his directorship and within his own department, must have been more practical and valuable.

## Dealing With Male Bosses, Father Figures, and Prestigious Pathologist Colleagues

As can be surmised from the above discussion, Abbott insightfully recognized the need to deal differently with each important male figure with whom she repeatedly interacted early in her career (Adami, Martin, Osler, Macphail, Keith, Warthin; Figure 2). She did not put the same effort into her interactions with Adami’s successor Horst Oertel, which unlike with these other men, commenced later in her career. Oertel had arrived in Montreal as head of pathology at MGH in early 1914, and, since she had no clinical duties, Abbott likely assumed their interactions to be unimportant. Although he became acting head at McGill during the War, Abbott expected Adami to return after its conclusion and so she simply ignored Oertel. Clearly, Abbott and Oertel did not like each other. Their disagreements were substantive, as their philosophies related to many aspects of pathology and medical education were diametrically opposed, as will be described in a later paper.

## Abbott’s Approaches in Comparison to Those of Other Contemporary Women Pathologists

In the early 20^th^-century, women academic “pathologists” were exceedingly rare; with the previously mentioned exception of Martha Wollstein who provided full anatomic and clinical pathology services at Babies Hospital in New York City, all others functioned as bacteriologists, experimental pathologists, or educators.^18,49-51^ One aspiring woman pathologist deserves particular mention. Dorothy Reed (1874–1964), a contemporary of Abbott who trained at Johns Hopkins and who co-discovered the Reed-Sternberg cell characteristic of Hodgkin’s Disease, took a strikingly different approach to dealing with male colleagues. According to Reed:

As long as I was in medicine I would never object to anything a fellow student or doctor did to me or in my presence if he would act or speak the same way to a man. If he were a bore, he would act like one—be loose in his conversation or jokes, slam a door in your face, hog the best of everything, be oblivious of any of the niceties of life or the courtesies—but if he discriminated against me because I was a woman—tried to push me around, was offensive in a way he wouldn’t be to a man, I would crack down on him myself—or take it up with the authorities if he proved too much for me alone. On the whole, this was the right way to take the position of women in medicine in the nineteenth century. It made life bearable, allowed me to make friends with some men who were not very pleasant persons—but knew no better, and earned me the respect and friendship of many of my associates. It didn’t endear me to one or two I fell afoul of, and undoubtedly I developed independence, even arrogance, which was foreign to my original nature. I was distinctly not such a “nice” person, but a stronger one after Johns Hopkins.^61(p72)^

While Reed claimed this more confrontational approach “served her well” and it may have felt gratifying in the short-term, it did not lead to career longevity. While Hopkins pathologist-in-chief William H. Welch (1850–1934) valued her work and repeatedly renewed her position as an apprentice, she noticed males promoted around her, and when she asked him why she had never been promoted, he explained “no woman had ever held a teaching position in the School and . . . there would be great opposition to it.”^61(p114)^ When Reed told Welch she was leaving, her biographer Peter Dawson concluded that Welch “seemed relieved” and “that the idea of the organized, domineering, and outspoken Dorothy as a permanent member of his department probably filled him with horror. He set to work at once to find her another position. She would have liked to stay in academic pathology, but few opportunities existed for women then. . .”^61(p116)^ Reed essentially lasted only 1 more year in pathology; while retraining in pediatrics at Babies Hospital, she worked as Wollstein’s assistant pathologist.^49,50^ Therefore, Reed’s final year of pathology practice was as a pediatric pathologist.

Clearly, Abbott’s less confrontational and more collaborative approaches allowed her to succeed in the masculine world of early 20th-century pathology. Reed, later Reed-Mendenhall, did not. She left pathology. After dealing with some health issues and the early loss of 2 of her 4 children, she joined the University of Wisconsin as a lecturer in its department of home economics, where she performed important research on infant mortality.^61^ It is undoubtedly safe to say that, had Abbott presented herself as “organized, domineering, and outspoken” and with expectations of being hired as a pathologist at a McGill teaching hospital, Abbott would not have had the same career trajectory.

One is hard-pressed to identify any other c1900 women pathologists,^18,49-51^ with the exception of Wollstein, to provide further comparisons. When Abbott was starting her career, she clearly did not have access to any practicing women pathologists to serve as mentors. I have found no evidence that Abbott and Wollstein were friends. Wollstein is only mentioned twice, on back-to-back days in June 1930, in Abbott’s personal diaries. Abbott hosted Wollstein, who was in Montreal for an American Pediatrics Association meeting, for dinner 1 night and for lunch the next day at the McGill’s Women’s University Club followed by providing a tour of her museum and its congenital heart collection (personal communication, Annmarie Adams, e-mails on August 20, 2023).^62^

Abbott’s less confrontational approach may also be foreshadowed in the words of her valedictorian speech; Abbott was the second woman graduate of McGill to have received this honor. The first was her close friend, Octavia Grace Ritchie (1868–1948), who graduated in 1888; Richie, ignoring explicit prior warning, “courageously defied official censorship of speech, and called for [McGill’s] doors of medicine to be opened.” Her speech was “met with cries of ‘Shame’ and ‘Never!’”^12(p184)^ Abbott’s speech, although she too dearly wanted to attend medical school at “patriarchal McGill,” ^12(p183)^ focused on the positive and exhorted women graduates to remember their “duties and privileges. . . at this early stage of women’s education in Canada ” and that “you, as members of the advance-guard, are in your own persons to be pointed out as instances of its success or failure.”^12(p183)^ While Richie and Abbott, who were both Bishop’s Medical School graduates, remained in Montreal, only Abbott ever held a faculty appointment at McGill. Ironically, in addition to the distinction of having been McGill valedictorians unable to gain admission to its own medical school, both now have McGill scholarships in their names.

## Abbott’s Retirement and Death

Abbott was notified in 1935 that she must retire the following year at the age 65 years^2,3^; she fought this unsuccessfully, but was told that it was McGill’s institutional policy. She could not invoke Dean Martin’s help this time as he too had turned 65 and was also required to retire. Once the outcome of her appeal was clear, she requested a promotion so that she could retire as professor emeritus, but this was also unsuccessful. Abbott retired in 1936^2,3^; presumably, because she was really born in 1868,^7^ she had actually worked at McGill until she was 68.

In March of 1936, just months before her retirement, Abbott broke another barrier and was granted membership to the previously all male McGill Faculty Club. Simultaneously, a Ladies’ Committee was formed to negotiate the ground rules as the new women members would only be granted access to a limited number of rooms.^14(p402)^

McGill awarded Abbott a second honorary degree, a LL.D (Legum Doctor) on October 22, 1936 (https://www.mcgill.ca/senate/files/senate/list_of_mcgill_honorary_degree_recipients_from_may_1935_to_present.pdf). This honor was bestowed not only to recognize her pioneering work in medical museology and medical history, but in the words of the citation, “above all as a stimulating teacher, an indefatigable investigator and a champion of higher education for women.”^14(p291)^ Abbott remained active in retirement with lecturing and writing.

In July of 1940, Abbott suffered a cerebral hemorrhage from which she did not recover. She lingered through the summer but died on Monday September 2, 1940 at the Montreal Neurological Institute. Her funeral service, which was held at the Christ Church Anglican Cathedral in Montreal on September 4, was attended by “hundreds of members of the medical profession and representative of outstanding Canadian families.”^63^ Both *The Daily Star* and *The Gazette* published a list of attendees, which included Martin as well as his successor as Dean of Medicine; her future biographer MacDermot, W.W. Francis, and many other recognizable names were on the list.^63,64^ She is buried along side her sister and grandmother at Christ Church Cemetery in Saint-Andre-d’Argenteuil, Laurentides Region, Quebec.^65^

English newspapers in Montreal ran stories honoring Abbott. For instance, on the day before her funeral, *The Gazette* included both a page 1 story^66^ and a more personal story on page 4 which begins as follows:

The death of Dr. Maude Elizabeth Seymour Abbott removes a pioneer in the fight for women’s equality in the professions. To her chosen field of medicine, and to her advocation of historical research, she brought talents which in themselves were an undeniable assertion of her right to their full exercise. . .^67^

This story describes the discrimination she had overcome during her lifetime in academics and medicine and notes that she was “never what is popularly known as a ‘feminist.’ And yet she did much to advance the emancipation of women from the shackles of all sorts of limitations and prohibitions from which they suffered.”^67^ It also highlighted her success as a medical historian, noting that she had been 1 of 3 physicians honored by the American Association of the History of Medicine with honorary membership, tangentially mentioning that 1 of the 2 others was Macphail.^67^

Obituaries written by male colleagues that were meant to honor her are dated and sexist. For instance, Abbott’s *CMAJ* obituary concludes with the following observation:

Doctor Abbott’s mind was characterized by three virtues, patience, perseverance, and versatility. While she often flitted from point to point in a way that was disconcerting to the mere male, yet she eventually came to order, and the final result was a piece of work logical, coherent, and worthwhile. Her magnetism was such that she got things done.^23(p395)^

W.W. Francis’s obituary explained her success in a less gendered manner as follows:

Like all of her activities, this [the IAMM] owed success largely to her indomitable energy and optimism, and to her genius for making friends with young and old and getting good work out of them. . . Always ready to do anything for others, short of abandoning one of her pet schemes, she was inclined to expect her friends. . . to do as much for her.^16(p306-307)^

As possibly the most extreme example of the latter, Francis notes that she once convinced a printer “to keep 130 pages of lead type standing (gratis) for 13 years”^16(p306)^ to facilitate the publication of Osler’s bibliography as a book, which she completed and published just shortly before her death.

Throughout life, because of her boundless energy, she had been called “the beneficent tornado.”^6(p156)^ While even supportive male friends sometimes considered her to be “impossible,” ^12(p193)^ they would have simultaneously concurred with Francis’s overall assessment: “For kindness of heart, exuberant enthusiasm, colossal industry, and sheer grit, she had no equal.”^16(p308)^

However, even Francis could not pass on an opportunity to poke fun at “her inveterate hero-worship which often prevented her seeing any defects in her elders and predecessors.”^16(p307)^ He noted this adversely affected her historical writings, such as her history of McGill, an institution she dearly loved, Francis noted: “Though blest with a ready sense of humor, it was characteristic of the ardent hero-worshipper that she never could understand why its title “McGill’s heroic past,” made some cynics snicker.”^16(p308)^

In closing, while Martin, Abbott’s closest colleague at McGill, once described her as “a feminine misfit in an exclusive male environment,” her accomplishments are impressive. She developed and utilized diverse strategies to run with the opportunities that were available to her in the masculine world of early 20th-century academic pathology. She became the foremost authority on medical museology, which played critical roles in the 19th and early 20th-century history of medicine. Just as knowledge of anatomy before it, knowledge of pathological anatomy underpinned the great advances in medical care that we have seen in the past century. Her pioneering work on CHD became the foundation for the fields of pediatric cardiology and corrective cardiovascular surgery. As a tribute to her life and amazing career and as an historical marker to an important transition point in the history of medicine, the Maude Abbott Medical Museum proudly lives on at McGill (https://www.mcgill.ca/medicalmuseum/introduction/history/physicians/abbott) and is open to the public.

Abbott’s success led others to consider how she managed. According to Gillett:

Although she worked for many years in an all male environment, Abbott did not – as others in her situation have sometimes tended to do – join the patriarchy and become condescending to members of her own sex. Among her closest friends were such avowed feminists as Drs. Grace Richie England and Carrie Derick, who worked for female franchise, birth-control, and social reform. Her own efforts at liberation were more specifically confined to matters of her own immediate concern. She had little time for more.^12(p191)^

Using the diverse strategies documented above, Abbott strove for personal success on her own terms, and she brought other women doctors along with her in her wake. Gillet also notes:

The strategies for survival are many. Becoming a “character,” embracing a mild form of eccentricity, is one of them – especially when, from the beginning, one is deemed to be deviant or different, and when the orthodox route to advancement is blocked. If Maude Abbott became slightly eccentric, it is no wonder. She worked in a very ambivalent professional world, where she was greatly admired but still remained “other.” She was both larger than life and lesser; world famous and a figure of fun.^12(p194)^

Unfortunately, Gillet’s sad analysis was accurate during her lifetime. Only after her death did Abbott finally become appropriately appreciated.

## Abbott’s Profound Impact on the Gender Demographics of Our Profession

While it is unfortunate that Abbott encountered discrimination throughout her life, her success, achieved as described above, promoted radical change in the pathology profession. The rare women physicians practicing 100+ years ago were expected by the medical profession and society to: (1) care for either women or children, (2) become religion-based missionaries to developing countries, (3) work as bacteriologists in public health laboratories, or (4) work as experimental pathologists doing animal-based research at medical schools under male department head supervision. These were the primary areas open to women physicians. Women physicians were not initially welcomed into the new developing field of hospital-based pathology.^18^ Martha Wollstein, the first North American pediatric pathologist, broke the barrier when she became the pathologist for Babies Hospital in New York City in the early 1890s; this happened because the hospital’s pediatrician-in-chief wanted an in-house pathologist and realized he could obtain Wollstein’s services inexpensively, as her family was independently wealthy and, as a single woman, she could live with her parents.^49,50^ Several decades later, Maud Menten followed in her footsteps in Pittsburgh.^51^ Between these 2 appointments, Abbott functioned as the CHD expert and medical museum curator at McGill. Although Abbott’s pathway into pathology was different, she was a pioneer, and of the 3, the only one who became famous while still alive. It should be a great source of pride to our subspecialty that the women physicians who opened the entire field of hospital-based pathology practice to women were pediatric pathologists. Furthermore, in many ways, these 3 women are also the true founders of pediatric pathology, as they pre-dated Sydney Farber or Edith Potter by several decades.

## Abbott’s Direct Contributions to Pediatric Pathology

Readers may be surprised to find out that, other than Abbott’s masterful chapters, reviews, and atlases on CHD,^24,60,68-75^ most of her publications have had little lasting significance to our profession. Appendix 2 in MacDermott’s biography^2^ lists Abbott’s publications; he reports a total of 122. Of these, ~30% are book reviews, editorials, and obituaries, and another ~30% are on medical/nursing history, Sir William Osler, or medical museology. Much of her original work on CHD related to these entities in adults; she also published regularly on acquired cardiovascular pathology. Her lasting accomplishments can be succinctly summarized as: (1) carefully studying and understanding CHD museum specimens, (2) providing statistics from her own experience and from the literature on types and frequencies,^24,76^ (3) observing how some types of CHD predispose to bacterial inflammatory processes, (4) networking with clinicians interested in CHD, (5) completing important collaborative clinic-pathological physiological studies with clinicians,^77,78^ and then (6) teaching the world through her beautifully illustrated chapters and atlases. Excellent reviews of Abbott’s CHD writings already exist.^79,80^ Abbott also published a few perinatal case reports, reflecting her interests in embryology and teratology.^81-84^

Late in her career, Abbott was a mentor to Montreal Children’s Hospital’s first pediatric pathologist, Frederick William Wiglesworth (1908–1985),^85^ who had just completed an internship in pathology at MGH. The hospital, then called Children’s Memorial Hospital, had been in existence since 1904 but its autopsy and surgical pathology cases, before his appointment in the spring of 1934, had always been handled by MGH. From 1934 to 1939, Wiglesworth reviewed museum and new CHD cases with Abbott.^4^ Abbott thought so highly of Wiglesworth that she left him many of her personal papers, which fortunately now reside in the Maude Abbott fonds at McGill (https://osler.library.mcgill.ca/media/pdf/p111.pdf ). Wiglesworth thought so highly of Abbott that he published his personal remembrances of her in *Perspectives in Pediatric Pathology*.^4^ Ironically, Wiglesworth served as president of the IAP (i.e., the IAMM successor organization) in 1960–61,^41^ an honor that eluded Abbott. Not surprisingly, Wiglesworth published extensively on CHD as well as many other pediatric pathology entities throughout his career. Wiglesworth has previously been recognized as a founder of pediatric pathology.^85^

Abbott’s lasting impacts on our profession were 2-fold and profound. First, while she was not the only CHD pioneer,^86^ Abbott made morphological and clinical information available and understandable to the medical profession, which opened doors to increasingly better treatments for CHD. She directly influenced pioneering pediatric cardiologist Helen B. Taussig (1898-1986) leading to future collaborative studies with Alfred Blalock (1899-1964) and together they pioneered corrective surgery.^87^ As attempts to surgically correct CHD became more commonplace, the need for post-mortem CHD morphological experts, a niche now typically filled by subspecialist pediatric pathologists, grew. To address this need, many of us, trained at institutions with large cardiac museum collections modeled after Abbott’s. Second, and more wholistically speaking, Abbott, in conjunction with Wollstein and Menten, founded pediatric pathology in North America.

## Supplemental Material

sj-pdf-1-pdp-10.1177_10935266241281786 – Supplemental material for Maude Abbott: “A Feminine Misfit in an Exclusive Male Environment” and Her Strategies for Success Supplemental material, sj-pdf-1-pdp-10.1177_10935266241281786 for Maude Abbott: “A Feminine Misfit in an Exclusive Male Environment” and Her Strategies for Success by James R. Wright in Pediatric and Developmental Pathology
