# Supplementary material for: Maude Abbott: “A Feminine Misfit in an Exclusive Male Environment” and Her Strategies for Success
Source: Pediatr Dev Pathol. 2024 Oct 1;27(6):513–29. doi: 10.1177/10935266241281786 (PMC11568640; doi:10.1177/10935266241281786)
Supplement: sj-pdf-1-pdp-10.1177_10935266241281786 – Supplemental material for Maude Abbott: “A Feminine Misfit in an Exclusive Male Environment” and Her Strategies for Success [file sj-pdf-1-pdp-10.1177_10935266241281786.pdf]

Invoice

James R. Wright, Jr.
Canada

Order ID: **D009762-U53414**

Order date: **28 Aug 2023**

Look and Learn Ltd
Office 370
19-21 Crawford Street
London W1H 1PJ
United Kingdom

Tel: +44 (0)20 7723 7732

support@lookandlearn.com

VAT reg: GB 849 1277 00

Item	Unit cost	Net		VAT
Image licence: Scholarly journal use, print and e-edition (hi-res)<br>*Project:* My selection (2023-08-28)<br>*Licensee:* James R. Wright, Jr.<br>*Images:* M589074	1 @ £50.00	£50.00	0%	£0.00
**Total**		**£50.00**		**£0.00**
**Paid by Visa**		**£50.00**		

Licensed images may be downloaded from: lookandlearn.com/downloads.

Images are supplied in accordance with the general Licensing Terms and Conditions and specific licence terms available at lookandlearn.com/downloads.

Registered in England with company number 5294671.
Registered office: Office 370, 19-21 Crawford Street, London W1H 1PJ, United Kingdom

Download licensed images

Selection: My selection (2023-08-28) (28 Aug 2023)

My selection (2023-08-28)

1

Granted	16:04 GMT on 28 Aug 2023
Project	My selection (2023-08-28)
Licensee	James R. Wright, Jr.
Licensed use	Scholarly journal use, print and e-edition (hi-res)
Full description	One-time inside use. Covers both print and e-editions, and any associated presentation or teaching use. Credit: Look and Learn. **Subject to Licensing Terms & Conditions.**
Licence term	*in perpetuity*
Cost (ex VAT)	£ 50.00 (1 image @ £ 50.00) Open receipt

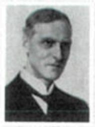

M589074: Sir Arthur Keith, Scottish anatomist and anthropologist

Download

W 3,422 px
H 4,676 px

Sir Arthur Keith (1866–1955), Scottish anatomist and anthropologist. Illustration for Outline of Modern Belief edited by JWN Sullivan and Walter Grierson (George Newnes, 1935).

Credit: Look and Learn

© 2005-2024 Look and Learn - All rights reserved
